# Prophylactic Use of Fluconazole in Very Premature Infants

**DOI:** 10.3389/fped.2021.726769

**Published:** 2021-10-01

**Authors:** Deshuang Zhang, Dongke Xie, Na He, Xiaoling Wang, Wenbin Dong, Xiaoping Lei

**Affiliations:** ^1^Division of Neonatology, Department of Pediatrics, The Affiliated Hospital of Southwest Medical University, Luzhou, China; ^2^Department of Pediatric Surgery, The Affiliated Hospital of Southwest Medical University, Luzhou, China; ^3^Sichuan Clinical Research Center for Birth Defects, Luzhou, China; ^4^Department of Perinatology, The Affiliated Hospital of Southwest Medical University, Luzhou, China

**Keywords:** nosocomial infection, fluconazole, prophylaxis, premature, infant

## Abstract

**Objective:** To evaluate the efficacy, safety, and fungal sensitivity of prophylactic fluconazole use in very premature infants.

**Methods:** We performed a retrospective historical comparative analysis of 196 very premature infants (113 in the prophylaxis group and 83 in the rescue group). The incidence of nosocomial fungal infection (NCFI) and pathogenic fungi, their drug sensitivity, and the minimum inhibitory concentration (MIC) of fluconazole were compared between the two groups. We also analyzed differences in short-term adverse outcomes, such as drug-induced liver or renal function disruption, fungal-attributable death, bronchopulmonary dysplasia (BPD), retinopathy of prematurity (ROP), periventricular leukomalacia (PVL), intraventricular hemorrhage (IVH), and necrotizing enterocolitis (NEC), between the groups. The effects of the prophylactic fluconazole strategy on NCFI and short-term adverse outcomes were assessed by multivariate logistic regression.

**Results:** *Candida albicans* (46.7%) and *Candida glabrata* (43.3%) were the main culprit pathogens causing NCFI. The incidence of NCFI was significantly lower in the prophylaxis group than in the rescue group (15.9 vs. 45.8%, *P* < 0.001). However, fewer fungi were completely sensitive to fluconazole (40 vs. 85%, *P* < 0.05) and the MIC of fluconazole was higher [16.0 (3.5 ~ 16.0) vs. 3.0 (1.0 ~ 8.0) μg/ml, *P* < 0.001] in the prophylaxis group than in the rescue group. Compared with the rescue group, the prophylaxis group had a lower risk of NCFI (adjusted OR 0.25; 95% CI 0.11, 0.55). Additionally, the prophylaxis group had significantly lower risks of combined outcomes (one or more complications, such as BPD, ROP needing interventions, PVL/IVH (grade > 2), NEC stage ≥2, and fungal-attributable death) (adjusted OR 0.44; 95% CI 0.21, 0.92). There was no significant difference in serum alanine transferase (ALT), aspartate transaminase (AST), creatinine (Cr), or direct bilirubin (DBIL) levels between the two groups.

**Conclusions:** Fluconazole prophylaxis reduced NCFI and improved combined clinical outcomes in very premature infants, with no increased risks of serious short-term adverse side effects; however, the MIC of fluconazole showed significant increases. Therefore, further optimization of preventive strategies is necessary to maintain the sensitivity of fluconazole against fungal isolates.

## Introduction

The survival rate of very premature and very low birth weight (VLBW, <1,500 g) infants has improved over the past several decades. Still, the immature immune systems of very premature infants (gestational age <32 weeks at birth), especially those with VLBW, frequent exposure to invasive procedures, use of broad-spectrum antibiotics, prolonged parenteral nutrition, and steroid treatment increase the risk of developing nosocomial fungal infection (NCFI) (1). Despite improvements in neonatal intensive care strategies and aggressive antifungal therapies, NCFI remains associated with high mortality and morbidity among very premature VLBW infants (2, 3). The mortality rate remains in excess of 25%, and approximately half of survivors may develop serious short- and long-term adverse sequelae, particularly neurodevelopmental impairment (NDI) (3–6). Many previous studies have reported that fluconazole prophylaxis has a positive effect on preventing fungal infection in preterm neonates (7–9). Prophylactic fluconazole is routinely used to prevent fungal infection in VLBW infants in the neonatal intensive care unit (NICU) (10, 11). Although multiple studies have indicated that fluconazole prophylaxis is effective and safe in preterm neonates (12, 13), such treatment is controversial (14, 15), especially regarding the emergence of resistance to antifungal agents. Thus, in our study, we aimed to evaluate the efficacy, safety, and drug sensitivity variation of prophylactic fluconazole for NCFI.

## Materials and Methods

The study was conducted in the Affiliated Hospital of Southwest Medical University, a tertiary-care hospital in southwest China. The protocol of the present study was reviewed and approved by the Institutional Research Ethics Committee of our institution. This retrospective analysis used only de-identified historical clinical data and did not require informed consent from the parents; however, consent from parents was taken at the time of treatment.

### Population Selection and Study Setting

This retrospective historical-comparative analysis included very premature infants with VLBW admitted to our institution within the first 24 h of life from April 1, 2016, to October 31, 2019. At first, 237 very premature infants with VLBW were included as potential subjects, out of which we excluded 41 subjects: fungal infection appeared within 2 days of life (*n* = 1), death occurred within 72 h of hospitalization (*n* = 3), serious congenital structural or chromosome anomalies (*n* = 6), hepatic insufficiency [alanine transaminase (ALT) or aspartate transaminase (AST) >3-fold the upper limit of normal, *n* = 11], and renal insufficiency [serum creatinine (Cr) > 1.5 mg/dL, *n* = 6] within 72 h of birth. Furthermore, 14 cases were excluded due to incomplete data. From April 1, 2016 to August 31, 2017, rescue treatment, not an antifungal prophylaxis strategy, was routinely used to address NCFI in very premature infants with VLBW in our NICU. In total, 83 cases were included in the rescue group. From September 3, 2017, all very premature infants with VLBW were routinely treated with an antifungal prophylaxis strategy. As of October 31, 2019, 113 infants who received antifungal prophylaxis were enrolled in the prophylaxis group of the study.

The clinical characteristics of the infants obtained from the medical records were gestational age, gender, birth weight, small for gestational age (SGA, birth weight below the 10th percentile at the same gestational age), multiple births, severe asphyxia (with asphyxia-related complications), birth in the hospital, endotracheal intubation, use of pulmonary surfactant (PS), early-onset sepsis (sepsis at ≤ 72 h of life), feeding intolerance (16), receipt of gastric tube and peripherally inserted central catheter (PICC), duration of gastric tube and PICC, use of broad-spectrum antibiotics, postnatal steroid use, days of parenteral nutrition and antibiotic therapy, and duration of hospital stay. The collected maternal data included maternal age, mode of delivery, premature rupture of membranes (PROM), meconium staining of amniotic fluid, antenatal corticosteroid use, antenatal antibiotic use, chorioamnionitis (diagnosis based on pathologic detection of the placenta), gestational diabetes mellitus or type II diabetes, and hypertensive disorders during pregnancy. Positive cultures from specimens of various sites and their sensitivity to antifungal agents were obtained, and the minimum inhibitory concentration (MIC) of fungal isolates was assessed. The susceptibility of *Candida* isolates to fluconazole was defined based on clinical breakpoints as follows (14): MIC ≤ 8 μg/ml as completely sensitive, MIC ≥64 μg/ ml as completely resistant, and MIC of 16–32 μg/ml as intermediately sensitive.

The blood cell counts, serum (1,3)-β-D-glucan levels, and chemical biomarkers of liver and renal function [ALT, AST, total bilirubin (TBIL), direct bilirubin (DBIL), and Cr] were monitored biweekly in our NICU. All data were obtained from the hospital information system.

### Definition of NCFI

Given the subtle, non-specific clinical features and difficulty of laboratory confirmation, there is still no optimal diagnostic criterion for fungal infection in premature infants (17). Therefore, isolating the fungal organism from a sterile site remains the gold standard for diagnosis, but it has a very low sensitivity rate (18). Thus, in VLBW infants with clinical manifestations, a culture of fungi from non-sterile sites was also used to diagnose fungal infection. Furthermore, in infants with clinical manifestations but negative cultures, laboratory tests [platelet counts, (1,3)-β-D glucan] and effective antifungal treatment were used to help with the diagnosis.

In our study, the definition of neonatal fungal infection included suspected diagnosis and confirmed diagnosis. (1) Suspected diagnosis was defined as infants with high-risk factors, clinical manifestations (such as hypothermia, poor cry or response, reluctance to feed, abnormal breathing, hypotension, bradycardia, abdominal distention), isolation of the fungal organism from a non-sterile site, and effective antifungal treatment (19) or infants with high-risk factors, clinical manifestations, positive laboratory findings [platelet counts <120^*^10E9/L, (1,3)-β-D glucan >125 pg/mL] (20, 21) and effective antifungal treatment. (2) Confirmed diagnosis was defined as infants with high-risk factors, clinical manifestations, and isolation of the fungal organism from a sterile site.

### Prevention and Treatment of NCFI

In the prophylaxis group, fluconazole was started intravenously on the 3rd postnatal day at a dose of 6 mg/kg twice a week for 4 weeks (in VLBW infants) and 6 weeks (in extremely low-birth-weight (ELBW) infants, <1,000 g at birth). The administration of fluconazole prophylaxis was suspended if the infant showed significant hepatotoxicity, needed full enteral feeding, was discharged from the hospital or died. In cases with a confirmed or suspected fungal infection in both groups, the rescue treatment strategy was given as an intravenous antifungal at a dose of 12 mg/kg. For suspected cases, empirical therapy was administered for 10–14 days; for confirmed cases, targeted treatment was continued for 14–21 days after the last positive blood culture (14, 22).

### Short-Term Outcomes

Data on the following short-term outcomes of the VLBW infants were obtained from the medical records: bronchopulmonary dysplasia (BPD), retinopathy of prematurity (ROP) needing interventions, periventricular leukomalacia (PVL) and intraventricular hemorrhage (IVH) grade >2, necrotizing enterocolitis (NEC) stage ≥2, and fungal-attributable death. BPD was diagnosed according to the NIH workshop criteria and the ROP definition was based on the international committee classification (23, 24). The definitions of PVL and IVH were based on cranial imaging, and the grade of IVH was determined according to Papile's classification (25). The modified Bell's stage criteria with clinical manifestations and abdominal X-ray have been used to diagnose NEC (26). Fungal attributable death was defined as the death of neonates who had clinical features of sepsis the week preceding the positive fungal culture, and no other pathogen was isolated from blood (7). Finally, combined outcomes were defined as the incidence of infants with at least one of the above five individual outcomes.

### Statistical Analysis

All the data of very premature infants with VLBW were recorded by two doctors using EpiData version 3.02 (The EpiData Association, Odense, Denmark), and all statistical analyses were performed with SPSS software, version 26.0 (SPSS Inc. Chicago, IL, USA).

Continuous variables are depicted as the means ± standard deviation (SD) or medians (interquartile range, IQR) and were compared between groups via Student's *t*-test or the Mann-Whitney U test. Categorical variables are depicted as the percentages (%) and were compared between groups via the chi-square test or Fisher's exact test. A multivariate forward step logistic regression with adjustment for important risk factors was used to calculate the adjusted odds ratio (OR) and 95% confidence interval (CI) for NCFI and short-term outcomes in the prophylaxis group relative to the rescue group. A two-sided *P* < 0.05 indicated statistical significance.

## Results

The baseline characteristics are summarized in Table 1, and there were no significant differences between the prophylaxis group and the rescue group.

**Table 1:** Comparison of infant baseline characteristics between two groups.

**Parameters**	**Rescue group (*n* = 83)**	**Prophylaxis group (*n* = 113)**	**χ^**2**^/*t***	***P***
**Neonatal characteristics**				
Gestational weeks, mean ± SD	29.8 ± 1.9	30.1 ± 1.6	−1.05	0.30
Birth weight, mean ± SD, kg	1.2 ± 0.2	1.3 ± 0.2	−1.20	0.23
Small for gestational age, *n* (%)	32 (39)	44 (39)	0.00	0.96
Male, *n* (%)	41 (49)	60 (53)	0.26	0.61
Multiple births, *n* (%)	27 (33)	39 (35)	0.08	0.77
Severe asphyxia, *n* (%)	15 (18)	23 (20)	0.16	0.69
Birth in our hospital, *n* (%)	70 (84)	85 (75)	2.40	0.12
**Maternal characteristics**				
Maternal age>35 years, *n* (%)	11 (13)	20 (18)	0.71	0.40
Vaginal delivery, *n* (%)	56 (67)	85 (75)	1.42	0.23
PROM>18 h, *n* (%)	35 (42)	42 (37)	0.50	0.48
Meconium staining of amniotic fluid, *n* (%)	3 (4)	2 (2)	0.12	0.73
Antenatal steroid use, *n* (%)	56 (67)	70 (62)	0.64	0.43
Antenatal antibiotic use, *n* (%)	41 (49)	52 (46)	0.22	0.64
Maternal chorioamnionitis, *n* (%)	5 (6)	9 (8)	0.27	0.60
Gestational diabetes mellitus, *n* (%)	8 (10)	8 (7)	0.42	0.52
Hypertensive disorders of pregnancy, *n* (%)	13 (16)	11 (10)	1.57	0.21

**Figure 1:**
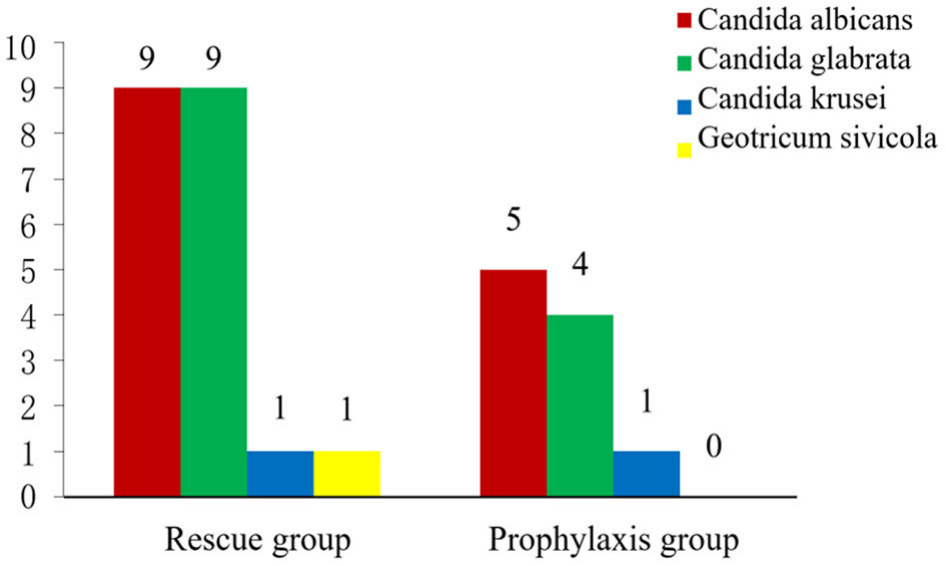
The distribution of fungal species between two groups.

Of the 196 very premature infants with VLBW during the study period, 38 in the rescue group and 18 in the prophylaxis group developed confirmed or suspected NCFI (45.8 vs. 15.9%, χ^2^ = 20.90, *P* < 0.001). Among the 56 infants with NCFI, 30 strains of fungi were isolated (20 strains in 14 patients in the rescue group and 10 strains in 7 patients in the prophylaxis group), which came from blood (*n* = 18), lower respiratory tract secretions (*n* = 7, 6 with positive blood culture), catheter tips of the PICC (*n* = 4, 3 with positive blood culture), and urine (*n* = 1). The distribution of fungal species is shown in Figure 1. The major isolated species were *Candida albicans* (14/30, 46.7%) and *Candida glabrata* (13/30, 43.3%). Isolates in the rescue group were more completely sensitive to fluconazole (85 vs. 40%, *P* < 0.05) and needed a lower MIC of fluconazole [3.0 (1.0 ~ 8.0) vs. 16.0 (3.5 ~ 16.0) μg/ml; *Z* = −11.14, *P* < 0.001) than those in the prophylaxis group. In addition to isolates that were completely sensitive to fluconazole, all others were intermediately sensitive but did not detect any isolates resistant to fluconazole. However, all isolated species were completely susceptible to amphotericin B, with a MIC ≤ 0.5 μg/ml in both study groups.

The possible risk factors for NCFI in both groups are presented in Table 2. Compared with infants in the rescue group, fewer infants in the prophylaxis group received broad-spectrum antibiotics, and these infants had a shorter duration of nasogastric tube usage, fewer days of antibiotic therapy, and a shorter hospital stay.

**Table 2:** Differences in risk factors for NCFI between two groups.

**Parameters**	**Rescue group (*n* = 83)**	**Prophylaxis group (*n* = 113)**	**χ^**2**^/*t*/*Z***	***P***
Endotracheal intubation, *n* (%)	70 (84)	95 (84)	0.00	0.96
PS use, *n* (%)	71 (86)	95 (84)	0.08	0.78
Early-onset sepsis, *n* (%)	33 (40)	34 (30)	1.99	0.16
Gastric tube placement, *n* (%)	81 (98)	106 (94)	0.82	0.37
Duration of gastric tube, mean ± SD, days	28.6 ± 17.4	23.2 ± 13.0	2.48	0.01
PICC use, *n* (%)	53 (64)	81 (72)	1.36	0.24
Duration of PICC, median (IQR), days	22 (0~37)	23 (0~33)	−0.24	0.81
Duration of parenteral nutrition, mean ± SD, days	30.5 ± 14.1	29.8 ± 10.0	0.39	0.70
Broad-spectrum antibiotic use, *n* (%)	63 (76)	68 (60)	5.34	0.02
Duration of antibiotic therapy, mean ± SD, days	28.4 ± 15.2	23.8 ± 11.1	2.33	0.02
Postnatal steroid use, *n* (%)	42 (51)	50 (44)	0.78	0.38
Duration of hospital stay, mean ± SD, days	45.8 ± 18.2	39.9 ± 12.4	2.55	0.01

Compared to those in the rescue group, infants in the prophylaxis group had a lower risk of NCFI (OR 0.25; 95% CI 0.11, 0.55), and the duration of antibiotic therapy was a risk factor for NCFI (OR 1.10; 95% CI 1.03, 1.17) (Table 3). At the same time, no obvious gastrointestinal side effects (vomiting, abdominal distension, or diarrhea) and no increased levels of ALT, AST, DBIL, or Cr in the prophylaxis group were observed (*P* > 0.05 for all) (Table 4). Furthermore, infants in the prophylaxis group had a lower risk of the combined clinical outcomes (OR 0.44; 95% CI 0.21, 0.92) than those in the rescue group (Table 5).

**Table 3:** The risks of NCFI in the prophylaxis group relative to the rescue group in the multivariate logistic regression model.

**Parameters**	**OR (95% CI)**	***P***
Duration of gastric tube (days)	1.03 (0.99 ~ 1.07)	0.14
Broad-spectrum antibiotic use	1.36 (0.35 ~ 5.24)	0.66
Duration of antibiotic therapy (days)	1.10 (1.03 ~ 1.17)	<0.01
Duration of hospital stay (days)	1.00 (0.95 ~ 1.06)	0.92
Prophylactic fluconazole therapy	0.25 (0.11 ~ 0.55)	<0.01

**Table 4:** Comparison of chemical biomarkers of liver and renal function between two groups.

		**ALT (U/L)**	**AST (U/L)**	**TBIL (μmol/L)**	**DBIL (μmol/L)**	**Cr (μmol/L)**
1st day	Rescue group	4.6 (3.6 ~ 7.3)	41.3 (31.1 ~ 61.9)	50.8 (42.7 ~ 61.7)	14.7 ± 5.0	51.9 (45.2 ~ 74.1)
	Prophylaxis group	4.9 (3.5 ~ 6.8)	43.8 (32.3 ~ 57.8)	48.7 (41.0 ~ 62.6)	15.9 ± 4.7	54.9 (48.5 ~ 64.1)
	*t*/*Z*	−0.15	−0.25	−1.02	−1.72	−0.73
	*P*	0.88	0.80	0.31	0.09	0.47
2nd week	Rescue group	5.7 (4.6 ~ 7.5)	19.6 (15.6 ~ 25.6)	71.9 ± 40.9	13.7 (11.1 ~ 17.4)	49.7 (40.0 ~ 55.8)
	Prophylaxis group	5.4 (3.8 ~ 7.0)	19.5 (15.8 ~ 25.0)	72.2 ± 40.3	15.0 (11.6 ~ 20.1)	50.0 (42.1 ~ 60.5)
	*t*/*Z*	−1.78	−0.33	−0.05	−1.50	−1.67
	*P*	0.08	0.74	0.96	0.13	0.10
4th week	Rescue group	8.2 (6.7 ~ 12.7)	23.4 (18.1 ~ 34.8)	34.6 (16.9 ~ 56.9)	13.4 (9.1 ~ 20.7)	41.6 ± 14.3
	Prophylaxis group	8.0 (5.8 ~ 12.8)	21.0 (17.0 ~ 28.0)	47.1 (21.7 ~ 69.1)	16.7 (10.1 ~ 27.1)	41.1 ± 11.8
	*t*/*Z*	−1.06	−1.92	−1.57	−1.64	0.30
	*P*	0.29	0.06	0.12	0.10	0.76

*Normally distributed data are presented as the means ± SD; nonnormally distributed data are presented as the medians (IQR)*.

**Table 5:** The incidences of short-term adverse outcomes between two groups.

**Adverse outcomes**	**Rescue group (*n* = 83)**	**Prophylaxis group (*n* = 113)**	OR^*^ (95% *CI*)	***P***
Combined outcomes^Δ^, *n* (%)	32 (39)	19 (17)	0.44 (0.21 ~ 0.92)	0.03
BPD, *n* (%)	27 (33)	15 (13)	0.48 (0.22 ~ 1.07)	0.07
ROP needing interventions, *n* (%)	5 (6)	3 (3)	1.12 (0.20 ~ 6.23)	0.90
PVL/IVH (stage > 2), *n* (%)	7 (8)	7 (6)	1.12 (0.34 ~ 3.64)	0.85
NEC (stage ≥ 2), *n* (%)	5 (6)	3 (3)	0.44 (0.09 ~ 2.17)	0.32
Fungal-attributable death, *n* (%)	3 (4)	2 (2)	1.90 (0.09 ~ 41.92)	0.69

Δ *Outcome with at least one case of BPD, ROP needing interventions, PVL/IVH (stage > 2), NEC (stage ≥ 2), fungal-attributable death*;

* *Adjusted for the duration of the gastric tube, broad-spectrum antibiotic use, duration of antibiotic therapy and hospital stays*.

## Discussion

NCFI is an increasingly frequent cause of late-onset infection in very premature infants. Due to its high mortality and morbidity, fungal infection is becoming a major concern in the NICU worldwide (18, 27). Fungal colonization is an important factor in fungal infections (28). The frequency of fungal colonization is up to 60% in VLBW infants during the 1st month of the hospital stay, and ~20% of these cases eventually progress to fungal infection (8). Preterm neonates have the highest risk of developing a fungal infection during the 2nd to 3rd week of life (28, 29). Consistent with these studies, our findings reveal that the median time to NCFI was the 19th day of hospitalization. The present study not only validated the efficacy of fluconazole prophylaxis in decreasing NCFI but also reminded us to pay attention to the decline in the sensitivity to fluconazole.

According to previously published literature (7–9, 30, 31), prophylactic antifungal medications effectively reduce fungal infection in preterm neonates. Our data also show that fluconazole reduced fungal infection in premature infants. In the present study, the prophylactic fluconazole strategy significantly decreased the incidence of fungal infection from 45.8% to 15.9% in very premature infants. These results are similar to those of a previous study (7), which showed a reduction from 43.2 to 21.0% after fluconazole prophylaxis. However, the incidences of fungal infection in a systematic review (27) and most other studies (6, 8, 9) were lower than those of our study in both study groups, especially in the rescue group. These discrepant results may be related to the low sensitivity for the diagnosis of fungal infection in these previous studies, which only used isolation of fungal organisms from sterile and/or non-sterile sites (7–9). Because of the low rate of positive fungal culture in clinical practice, most fungal infections might be ignored in these studies.

*Candida albicans* and *Candida glabrata* were the predominant fungal pathogens and accounted for 46.7 and 43.3% of the total number of isolated fungal germs, respectively. However, in previous studies (14, 32), *Candida albicans* and *Candida parapsilosis* were the most commonly identified species of fungal infection in preterm infants. This change in the distribution of *Candida* species may be related to hospital-specific disparities. Furthermore, these results may highlight a shift in *Candida* species causing fungal infection in recent years.

In the present study, compared to the rescue period, the complete sensitivity rate to fluconazole in the fluconazole prophylaxis period decreased from 85 to 40%, and the MIC of fluconazole increased from 3.0 [1.0 ~ 8.0] to 16.0 [3.5 ~ 16.0] μg/ml. However, even though the sensitivity to fluconazole was decreased, no completely resistant fungi were isolated. Thus, after exposure to a low dosage of fluconazole, *Candida* species might act in concert, leading to stepwise increases in MIC and broadening of the azole resistance spectrum (29, 30). In addition, *Candida glabrata* was the 2nd most frequent species in our study, and it is more prone to developing fluconazole resistance than other *Candida* species (31), which might explain the decreased fungal sensitivity to fluconazole in the prophylaxis group. Although previous studies showed no significant differences in drug resistance (30, 33, 34), fluconazole-resistant strains are reported sporadically (7, 12, 33), and our finding further confirmed this trend of drug resistance by the change in MIC.

Another finding in our study is that prolonged antibiotic use was a risk factor for NCFI, which is consistent with other studies (34). Broad-spectrum antibiotics (third-generation cephalosporins or carbapenems) are commonly used to treat bacterial sepsis in our NICU. Premature infants are prone to early colonization by fungi due to an immature immune system and poor skin and mucosal barriers (35). However, prolonged use of broad-spectrum antibiotics may lead to fungal infections by suppressing the normal flora and allowing fungi to occupy muco-epithelial niches that facilitate invasion and dissemination (34). Thus, prophylactic fluconazole and shortened use of antibiotics are all important strategies for reducing NCFI.

Fungal infection is attributable to increasing mortality and NDI in premature infants. A previous study reported that (35), among those with late-onset infection, preterm infants with *Candida* infection had the highest risks of death and/or NDI. In the present study, no significant differences in BPD, ROP, PVL/IVH, NEC, or fungal-attributable death were observed between the two groups, consistent with previous reports (8, 9, 30, 36). However, the BPD showed a trend toward a lower level, at 13%, in the prophylaxis group compared to 33% in the rescue group, but it did not reach statistical significance. Because the rate of each outcome was very low and the sample size small, we combined the outcomes related to fungal infection to improve the statistical power. We found that the combined outcomes were improved during the prophylaxis period, which was very likely attributed to reductions in fungal infection, one of the most important risk factors for BPD (37). This finding will be further confirmed in large-scale studies in the future. Side effects are another major concern for the prophylactic use of fluconazole in VLBW infants. Consistent with previous literature (7, 9, 37), there were no significant differences in gastrointestinal adverse reactions, hepatic toxicity, renal toxicity, or cholestasis between the two groups.

In this study, the fungal infection rate in the placebo group was much higher than that in the Benjamin paper group (9). Benjamin et al. (9) included only the cases with a positive culture. Because the sensitivity of fungal cultures was shown to be very low in clinical practice (18), this study also considered clinically suspected fungal infections with negative cultures. When using the same criteria as Benjamin et al. (9), the overall incidence of fungal infection was 16.9% (14/83) in the placebo group (20 positive cultures from 14 patients).

Some limitations of this study should be considered. First, while we minimized confounding factors as much as possible, the duration of antibiotic exposure remained imbalanced in both groups, which might weaken the effect power of prophylactic fluconazole. Additionally, this was a retrospective historical comparative analysis. Practice changes such as ongoing advances in neonatal care, continuous improvement in perinatal care, and advancing strategies in antimicrobial stewardship, might have contributed to the results. In addition, the dosages and courses may have different results with prophylactic fluconazole. Despite these shortcomings, we found significant increases in MIC during the period of prophylactic fluconazole, which was likely attributed to the widespread emergence of *Candida glabrata*, a fungus susceptible to developing fluconazole resistance (38).

## Conclusions

In conclusion, fluconazole prophylaxis reduced NCFI and improved combined clinical outcomes in very premature infants, with no increased risks of serious short-term adverse side effects; however, the MIC of fluconazole showed significant increases. Therefore, further optimization of preventive strategies is necessary to maintain the sensitivity of fluconazole against fungal isolates.

## Data Availability Statement

The original contributions presented in the study are included in the article/supplementary material, further inquiries can be directed to the corresponding author/s.

## Ethics Statement

The studies involving human participants were reviewed and approved by Institutional Research Ethics Committee of The Affiliated Hospital of Southwest Medical University. Written informed consent from the participants' legal guardian/next of kin was not required to participate in this study in accordance with the national legislation and the institutional requirements.

## Author Contributions

DZ: data processing, statistical analysis, and writing papers. DX, XW, and NH: data collection and management. WD: research conception and design. XL: article revision and quality control, overall responsibility for the article, supervision, and management. All authors contributed to the article and approved the submitted version.

## Funding

This work was partly supported by the Sichuan Science and Technology Program, Grant No. 2019YJ0696 to XL. The funding body had no role in the design of the study and collection, analysis, and interpretation of data or in writing the manuscript.

## Conflict of Interest

The authors declare that the research was conducted in the absence of any commercial or financial relationships that could be construed as a potential conflict of interest.

## Publisher's Note

All claims expressed in this article are solely those of the authors and do not necessarily represent those of their affiliated organizations, or those of the publisher, the editors and the reviewers. Any product that may be evaluated in this article, or claim that may be made by its manufacturer, is not guaranteed or endorsed by the publisher.
